# Blood Amyloid-β Reduction Following Chongnoi-tang Treatment in Alzheimer’s Spectrum Conditions: A Retrospective Pilot Observational Study

**DOI:** 10.3390/jcm15187329

**Published:** 2026-09-21

**Authors:** Jinhyuk Lee

**Affiliations:** Chongnoi Korean Medical Clinic, Seoul 06541, Republic of Korea; jhlee@i-chn.net; Tel.: +82-2-518-7535

**Keywords:** amyloid beta-peptides, biomarkers, Alzheimer’s disease, apolipoprotein E4, drugs, Chinese herbal

## Abstract

Background/Objectives: Anti-amyloid immunotherapies reduce amyloid-β (Aβ) plaques but carry a 15–35% risk of amyloid-related imaging abnormalities (ARIA), particularly in APOE ε4/ε4 carriers. This preliminary observational study evaluated blood Aβ oligomer changes following treatment with the multi-component herbal formula Chongnoi-tang (CNT) and contextualized these findings against natural disease trajectories in National Alzheimer’s Coordinating Center (NACC) and Alzheimer’s Disease Neuroimaging Initiative (ADNI) reference cohorts. Methods: This retrospective pilot cohort included 16 patients with Alzheimer’s spectrum conditions treated with CNT. Blood Aβ oligomers were measured using the AlzOn Plus immunoassay. Primary analysis (*n* = 13) excluded cases with a ceiling-level baseline value, an intercurrent potentially confounding condition, or no true baseline measurement. Results: Mean blood Aβ oligomer levels decreased by 15.8% ± 13.4% at 6 months (median −20.6%; 95% CI −23.9% to −7.8%; Wilcoxon signed-rank test, *p* < 0.001; paired Cohen’s dz = 1.07). At each participant’s final available follow-up, all 13 primary-analysis patients had values below baseline. High-risk patients (*n* = 4) showed a mean 25.7% reduction. Baseline Aβ level and 6-month percent biomarker change exhibited an exploratory, non-significant inverse trend in the Pearson correlation (r = −0.489, *p* = 0.090) that was weaker and non-significant in the Spearman sensitivity analysis (ρ = −0.300, *p* = 0.320), consistent with regression to the mean and mathematical coupling as plausible contributors. Importantly, all four APOE ε4/ε4 homozygotes showed Aβ reductions without ARIA-like clinical events. Mini-Mental State Examination (MMSE) worsening occurred in 37.5% (3/8) of CNT-treated patients versus 42.9% (15/35) in propensity-matched NACC controls (*p* = 0.89); given the small sample, this comparison is inconclusive. Conclusions: In this small, uncontrolled pilot study, CNT was associated with blood Aβ reductions, which should be interpreted as a statistical association rather than confirmed evidence of a treatment effect; no treatment discontinuations due to adverse effects were documented in this small cohort, including among APOE ε4/ε4 carriers. These hypothesis-generating findings warrant further confirmation in prospective randomized controlled trials.

## 1. Introduction

Alzheimer’s disease (AD) is a progressive neurodegenerative disorder and the most common cause of dementia worldwide, affecting approximately 55 million individuals, and is projected to affect 139 million individuals by 2050 [1]. Despite decades of research effort, disease-modifying therapeutic options remain limited. The approval of anti-amyloid monoclonal antibodies, such as lecanemab and donanemab, has provided clinical evidence that amyloid lowering can modestly slow cognitive and functional decline in selected patients with early AD [2,3].

Despite this clinical milestone, broad implementation of anti-amyloid immunotherapies is constrained by the risk of amyloid-related imaging abnormalities (ARIA), encompassing cerebral edema (ARIA-E) and microhemorrhage (ARIA-H), which occur in 15–35% of treated patients and are substantially more frequent and severe in APOE ε4/ε4 carriers [2,3,4]. This safety profile poses a particularly important safety and monitoring challenge in APOE ε4/ε4 carriers, who carry the highest genetic risk. Additionally, BACE1 inhibitors failed uniformly across clinical trials, owing to cognitive worsening attributable to BACE1’s role in processing neurophysiologically essential substrates [5].

These limitations have motivated the investigation of alternative therapeutic strategies that target the multiple pathways implicated in AD pathophysiology, including Aβ production and clearance, tau phosphorylation, neuroinflammation, and cholinergic dysfunction. Multi-component herbal formulas are, in principle, capable of engaging several of these pathways simultaneously, a pharmacological framework described in network pharmacology approaches to traditional medicine [6]; however, whether any specific multi-herb formula achieves clinically relevant multi-target activity requires empirical demonstration rather than assumption. Chongnoi-tang (CNT) is a multi-herb formula used in routine clinical practice at the authors’ clinic for patients with cognitive decline; whether it exerts genuine multi-target pharmacological activity has not been established and is not directly tested here but was evaluated in this pilot cohort for its association with a peripheral blood Aβ oligomer biomarker.

Blood-based biomarkers of Aβ pathology have drawn interest as a less invasive alternative to cerebrospinal fluid or amyloid PET measures for tracking disease-related change in routine clinical settings [7]. The AlzOn Plus Multimer Detection System used in this study quantifies Aβ oligomer self-aggregation propensity, a biomarker readout that is mechanistically distinct from, and not established as directly interchangeable with, the plasma Aβ42/40 ratio assays used in large research cohorts such as the Alzheimer’s Disease Neuroimaging Initiative (ADNI) (see Section 2.3).

This brief report presents preliminary real-world pilot data evaluating blood Aβ oligomer dynamics in patients with Alzheimer’s spectrum conditions receiving CNT, contextualized against natural history trends from National Alzheimer’s Coordinating Center (NACC) and ADNI registries.

## 2. Materials and Methods

### 2.1. Study Design and Participants

This retrospective observational pilot study was conducted at the Chongnoi Korean Medical Clinic, Seoul, Republic of Korea. All clinical data used in this study were generated during ordinary patient care as part of each patient’s routine CNT treatment and follow-up, beginning 22 July 2023; this care was not conducted as part of a research protocol and preceded IRB review. The retrospective research protocol under which these pre-existing clinical records were extracted and analyzed for research purposes was subsequently approved by the KoNIBP Public Institutional Review Board (Public IRB, Exemption No. P01-202603-01-052; date of approval: 18 March 2026), and the study was conducted in accordance with the Declaration of Helsinki. Extraction and analysis of the pre-existing clinical records for research purposes occurred only after this approval date; clinical follow-up continued as part of ordinary care after approval, through the final study assessment on 18 May 2026, with no study-specific intervention or additional data collection procedure introduced beyond routine clinical practice. The requirement for informed consent for the retrospective research use of pre-existing clinical records was waived by the IRB, given the retrospective observational design without additional investigational intervention. Written informed consent for the publication of anonymized individual data was separately obtained from all participants.

Eligible participants were adults with Alzheimer’s spectrum conditions: (1) asymptomatic with borderline-or-above blood Aβ (cognitively normal with blood Aβ ≥ 0.78 ng/mL on AlzOn Plus assay; this is the assay manufacturer’s borderline risk threshold, identical to the threshold defined in Section 2.3); (2) mild cognitive impairment (MCI) per National Institute on Aging-Alzheimer’s Association (NIA-AA) criteria [8]; or (3) early-onset dementia (symptom onset before age 65). Participants were included if they had at least one baseline blood Aβ measurement prior to CNT initiation and at least one follow-up measurement after ≥6 months. Three patients were excluded from the primary statistical analysis: P8 (newly diagnosed hypothyroidism identified at the 6-month assessment, untreated at the time of blood sampling), P14 (absent true baseline), and P10 (ceiling-level baseline of 5.00 ng/mL precluding reliable absolute quantification). The primary analysis cohort comprised 13 patients.

### 2.2. Intervention and Quality Consistency

**Figure 1:**
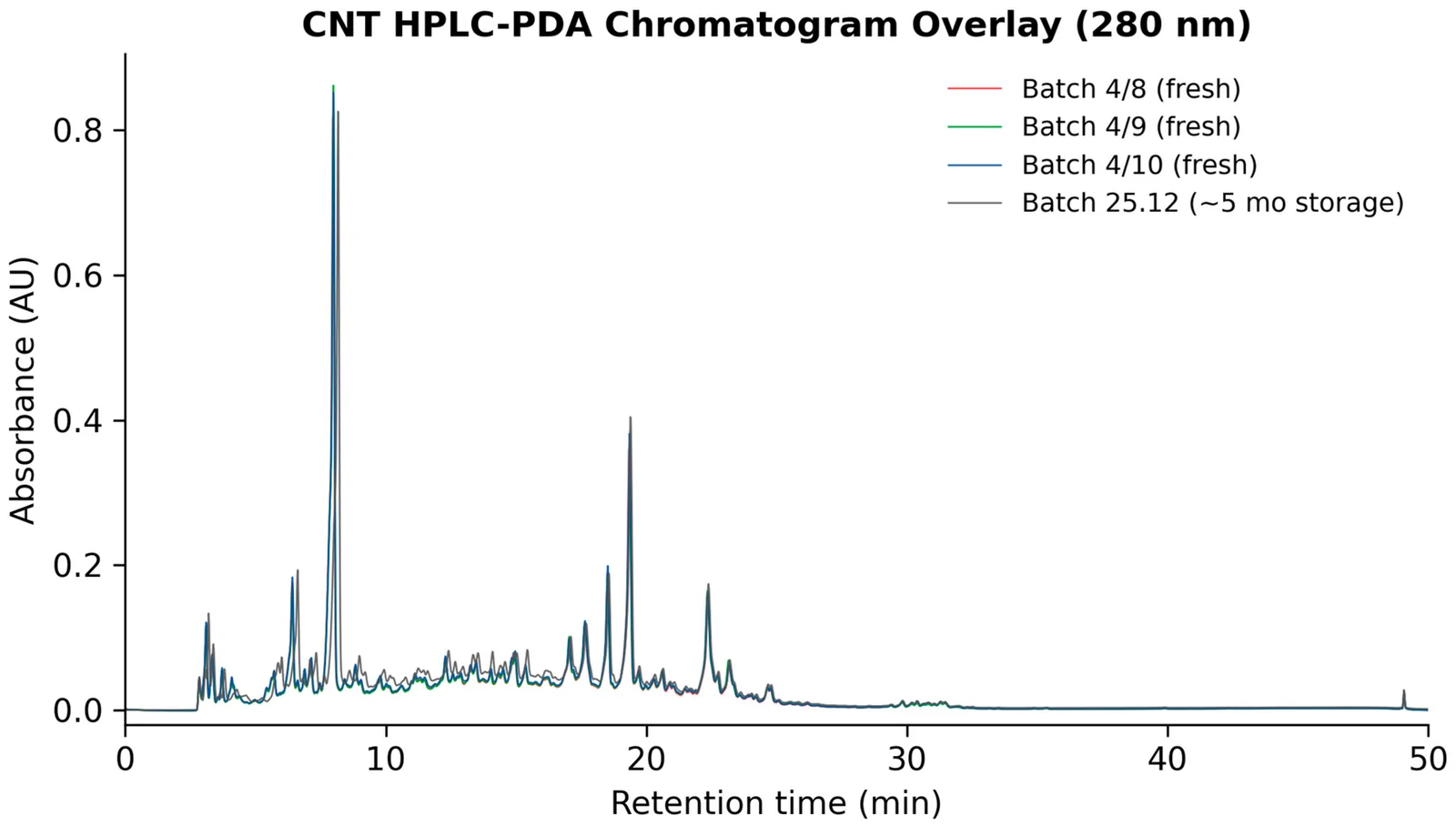
Overlay HPLC-PDA chromatograms of Chongnoi-tang (CNT) production batches, including three recently prepared production batches (4/8, 4/9, 4/10, decocted within the same week) and one batch (25.12) stored at room temperature for approximately 5 months prior to analysis, monitored at 280 nm. Peak patterns and relative peak-area ratios were consistent across all four samples, indicating both batch-to-batch reproducibility and preserved chemical composition after prolonged ambient storage.

CNT was administered as a standardized oral preparation (typically three doses daily). CNT is composed of sixteen botanical components: Dimocarpus longan Lour. (arillus), *Rehmannia glutinosa* (Gaertn.) DC. (radix), *Platycladus orientalis* (L.) Franco (semen), *Crataegus pinnatifida* Bunge (fructus), *Salvia miltiorrhiza* Bunge (radix), *Poria cocos* (Schw.) Wolf (sclerotium), *Angelica gigas* Nakai (radix), *Glycyrrhiza uralensis* Fisch. (radix), Magnolia officinalis Rehder & E.H.Wilson (cortex), *Polygala tenuifolia* Willd. (radix), *Acorus gramineus* Aiton (rhizoma), *Ziziphus jujuba* Mill. var. spinosa (semen), *Citrus unshiu* Marcow. (pericarpium), *Rosa roxburghii* Tratt. (fructus), *Lycium chinense* Mill. (fructus), and *Nelumbo nucifera* Gaertn. (semen). CNT is prepared by decoction of these sixteen botanical components in an aqueous ethanol solvent (approximately 3% ethanol) under standardized time–temperature conditions at a licensed traditional Korean medicine pharmacy; the low ethanol concentration is used as a co-solvent during the initial extraction rather than added afterward. Each oral dose consisted of one packaged 30 mL unit of decoction administered orally, typically three times daily; quantitative botanical mass ratios and per-herb dosages remain proprietary pending intellectual property proceedings. To assess both batch-to-batch consistency and storage stability, four samples of CNT were analyzed by HPLC with photodiode array detection (Waters 2695 HPLC system with 2996 PDA detector; Waters Corporation, Milford, MA, USA; Empower 3 software): three recently prepared production batches (4/8, 4/9, 4/10) and one batch (25.12) was stored at room temperature for approximately 5 months prior to analysis. Overlay comparison at 280 nm showed consistent peak patterns and relative peak-area ratios, indicating batch reproducibility and composition preservation (Figure 1). This HPLC-PDA fingerprint provides an objective, reproducible quality-control benchmark that is independent of the proprietary formulation details; the authors intend to disclose the complete quantitative formulation following finalization of pending patent applications to enable full external replication. Individual dosing frequency, treatment duration, dose modifications, and concomitant medications for each patient are summarized in Table 1.

### 2.3. Biomarker Assay and Contextual Comparator Data

Blood Aβ oligomers were quantified using the AlzOn Plus immunoassay (PeopleBio Inc., Seoul, Republic of Korea; MFDS Approval No. 체외제허 18-289; CE marking under the EU In Vitro Diagnostic Regulation [IVDR] 2017/746) [7]. The assay employs a Multimer Detection System (MDS) measuring Aβ oligomer self-aggregation propensity. Risk categories, as defined by the assay manufacturer were as follows: low-risk (≤0.77 ng/mL), borderline (0.78–0.92 ng/mL), and high-risk (≥0.93 ng/mL). These categories reflect assay-defined blood Aβ oligomer strata and are not a validated measure of clinical AD risk per se. A direct inquiry to the assay manufacturer, conducted in September 2026, prompted by peer review questions on this point, yielded an updated within-run coefficient of variation (CV) estimate of ~15%, which is used throughout this manuscript in place of the manufacturer’s earlier published specification of ~10%.

For exploratory contextual reference only, two independent comparator sources were referenced; neither served as a concurrent or randomized control group. Disease-trajectory comparators from the National Alzheimer’s Coordinating Center (NACC) database [9] were propensity-score matched to the CNT cohort’s paired-MMSE subset (*n* = 8) using 1:5 nearest-neighbor matching (up to five NACC matches per CNT case) on age, sex, baseline MMSE, APOE ε4 status, and exact diagnostic category, with a caliper of 0.05 standard deviations, yielding 35 matched NACC comparators (one of the eight CNT cases did not have five eligible matches within the caliper); all post-matching standardized mean differences were <0.1, indicating adequate covariate balance. Separately, untreated MCI patients from the Alzheimer’s Disease Neuroimaging Initiative (ADNI) database were referenced descriptively; all ADNI participants meeting the same MCI diagnostic criteria, untreated status, comparable age and sex distribution, and a comparable education-year band to the CNT cohort’s MCI subgroup, with available baseline and 12-month plasma Aβ42/40 [Lumipulse platform] measurements, were included without further selection (*n* = 41). Because the CNT cohort’s blood Aβ oligomer levels (AlzOn Plus, Multimer Detection System) and ADNI’s plasma Aβ42/40 (Lumipulse platform) reflect distinct analytes and assay methodologies that are not interconvertible, the ADNI comparison is presented only as a brief, qualitative, directional, natural history context and should not be interpreted as a quantitative benchmark of treatment effect. Blood samples in the CNT cohort were drawn during routine clinic visits without a fixed, pre-specified time-of-day or fasting protocol; potential physiological variation in circulating Aβ oligomer levels related to sampling conditions cannot be excluded (see Limitations).

### 2.4. Statistical Analysis and Generative AI Disclosure

The primary outcome was a percent change in blood Aβ oligomers from baseline to 6 months, summarized as the mean, standard deviation, median, and 95% confidence interval (t-distribution), together with the paired-sample effect size (Cohen’s dz, calculated as the mean of the paired absolute [ng/mL] baseline-to-6-month differences divided by the standard deviation of those paired differences). Paired pre-post comparisons used the Wilcoxon signed-rank test. Pearson correlation assessed the relationship between baseline Aβ and percent change; because percent change is mathematically derived in part from the baseline value, and because regression to the mean can independently generate an inverse baseline-change relationship even in the absence of any treatment effect, a Spearman rank correlation was additionally performed as a sensitivity analysis; both coefficients are interpreted cautiously. Two-tailed *p* < 0.05 was considered statistically significant. For the exploratory MMSE comparison, worsening was defined as any decline in MMSE score from baseline to follow-up, without a pre-specified minimum clinically meaningful threshold, given the exploratory nature of this analysis; this definition should be interpreted with caution, since it captures even a one-point decline. All statistical analyses were performed using Python 3.12 (SciPy 1.17 and NumPy 2.4).

Generative AI Disclosure: During manuscript preparation, Claude Sonnet 5 (Anthropic, San Francisco, CA, USA) was used for English-language refinement, drafting of statistical analysis code, and organization of literature references. The author reviewed and verified all AI-assisted content and analyses, and takes full responsibility for all content, analyses, and conclusions in this manuscript.

## 3. Results

### 3.1. Patient Characteristics and Primary Outcome

**Figure 2:**
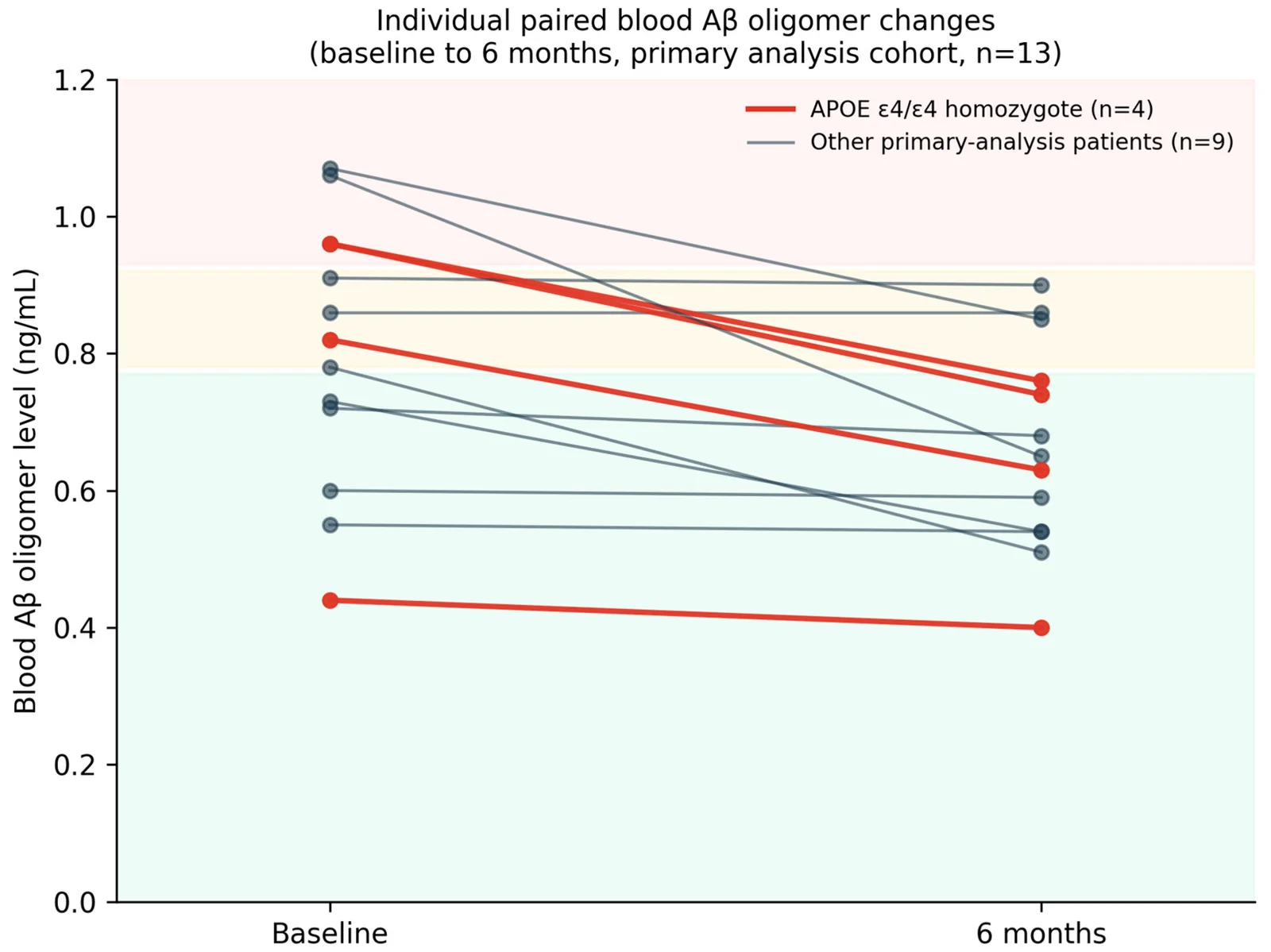
Individual paired blood Aβ oligomer changes from baseline to 6 months in the primary analysis cohort (*n* = 13). Each line connects one patient’s baseline and 6-month value; red lines indicate APOE ε4/ε4 homozygotes (*n* = 4) and grey lines indicate other primary-analysis patients (*n* = 9). Shaded horizontal bands denote the AlzOn Plus assay’s manufacturer-defined risk categories (green, low-risk ≤ 0.77 ng/mL; yellow, borderline 0.78–0.92 ng/mL; red, high-risk ≥ 0.93 ng/mL; Section 2.3). This figure is provided in response to reviewer comments requesting graphical presentation of individual paired changes.

Sixteen patients were enrolled (median age 63 years, range 49–84; 4 APOE ε4/ε4 homozygotes) (Table 2). In the primary analysis cohort (*n* = 13), mean blood Aβ decreased from 0.80 ± 0.19 ng/mL at baseline to 0.67 ± 0.15 ng/mL at 6 months, representing a mean reduction of 15.8% ± 13.4% (median −20.6%; 95% CI −23.9% to −7.8%; Wilcoxon signed-rank test, *p* < 0.001; paired Cohen’s dz = 1.07). Individual paired baseline-to-6-month changes are shown graphically in Figure 2. All 13 primary-analysis patients demonstrated reduction from baseline at final follow-up. P10 (ceiling baseline 5.00 ng/mL) decreased to 1.40 ng/mL at 6 months and 0.71 ng/mL at 12 months (reported descriptively). In a sensitivity analysis including P8 (excluded from the primary analysis because of newly diagnosed hypothyroidism at 6 months), the overall direction of change remained toward lower blood Aβ oligomer levels (*n* = 14: mean −12.9% ± 16.9%, median −14.9%; Wilcoxon signed-rank test, *p* = 0.013).

### 3.2. Subgroup Analyses, Risk Transitions, and APOE ε4/ε4 Safety

High-risk patients (*n* = 4) showed a mean reduction of 25.7% at 6 months. Borderline patients (*n* = 4) showed 14.7%, and low-risk patients (*n* = 5) showed 8.8%. A negative trend was observed between the baseline Aβ level and 6-month percent biomarker change on the Pearson correlation (r = −0.489, *p* = 0.090); a Spearman rank correlation performed as a sensitivity analysis was weaker and not statistically significant (ρ = −0.300, *p* = 0.320), consistent with the possibility that this pattern partly reflects mathematical coupling between baseline values and change scores, or regression to the mean, rather than a true dose-baseline relationship. At 6 months, six of thirteen patients moved to a lower AlzOn Plus risk category (as defined in Section 2.3) and none moved to a higher category; because these categories reflect assay-based blood Aβ oligomer strata rather than a validated measure of AD risk itself, this should be interpreted as a biomarker-level observation, not evidence of reduced clinical AD risk. By 12 months, one patient (P3) showed risk category deterioration after a dose reduction; the clinical significance of this temporal association is uncertain and cannot establish causality from a single observation.

Importantly, all four APOE ε4/ε4 homozygotes (P3, P5, P9, P13) demonstrated Aβ reductions at 6 months (−22.9%, −20.8%, −23.2%, −9.1%) without any ARIA-like neurological adverse clinical events (routine MRI surveillance was not performed).

### 3.3. Reference Cohort Comparison and Safety

In the subset with paired MMSE (*n* = 8; individual patient trajectories presented in Table 3), MMSE worsening—defined as any decline in MMSE score from baseline without a pre-specified minimum threshold—occurred in 3/8 (37.5%) CNT-treated patients, compared with 15/35 (42.9%) in the PSM-matched NACC reference group (Section 2.3; *p* = 0.89); given the small sample size on the CNT side and the resulting wide overlap, this comparison should be regarded as inconclusive rather than as evidence of a difference in cognitive trajectory. Untreated ADNI MCI controls (*n* = 41) showed longitudinal worsening in plasma Aβ42/40 over 12 months; because this reflects a different biomarker and assay platform from the CNT cohort’s blood Aβ oligomer measurement, this is noted only as a brief, qualitative context and is not a quantitative treatment comparison. No patient discontinued treatment due to adverse effects and no ARIA-like clinical events occurred; routine MRI surveillance for subclinical ARIA was not performed in this cohort.

## 4. Discussion

This brief report provides preliminary, hypothesis-generating observational evidence of an association between CNT treatment and blood Aβ oligomer reductions across the Alzheimer’s spectrum; these uncontrolled, retrospective data do not establish causality, disease modification, or therapeutic efficacy.

The inverse trend between baseline Aβ level and percent change (Pearson r = −0.489, *p* = 0.090; Spearman ρ = −0.300, *p* = 0.320) was exploratory and not statistically significant on either measure. Regression to the mean and mathematical coupling between baseline values and change scores are plausible, and in our assessment, likely material, contributing explanations, particularly given the weaker, non-significant Spearman correlation obtained in sensitivity analysis. Any interpretation involving a homeostatic mechanism or enhanced Aβ clearance remains speculative, as clearance itself was not directly measured in this study; restorative physiological pathways, such as glymphatic efflux [10], LRP1-mediated blood–brain barrier transport [11], and microglial phagocytosis [12], represent untested hypotheses for future mechanistic study rather than established explanations. Using the updated ~15% within-run coefficient of variation obtained directly from the assay manufacturer (Section 2.3), and assuming independent measurement error of similar magnitude at both time points, the approximate analytical variability of a single patient’s baseline-to-6-month percent difference would be on the order of 21% (√2 × 15%), even in the complete absence of any biological change. Several individual percent changes, including some considerably larger than the smallest values (0%, −1.1%, −1.7%, and −1.8%; Table 2), fall within this range, and individual-level changes of this magnitude should, therefore, not be interpreted as evidence of a biological treatment effect in a given patient. The group-level finding is, nonetheless, more robust than any single individual value because it does not rely on the magnitude of change exceeding assay noise: twelve of the thirteen primary-analysis patients showed a decrease at 6 months, and the remaining patient showed no change (0.0%), with none showing an increase. This highly consistent directional pattern would be expected to arise from symmetric measurement noise alone only infrequently (Wilcoxon signed-rank test, *p* < 0.001, reflects this directional consistency rather than the magnitude of any individual value). More broadly, a reduction in this circulating biomarker should not be equated with reduced cerebral amyloid burden or disease modification, as the relationship between blood Aβ oligomer levels and brain amyloid pathology was not established in this cohort (see Limitations). Throughout this manuscript, statistical significance (*p*-values), analytical variability (assay CV), biological significance, and clinical significance are conceptually distinct; a statistically significant group-level change does not, by itself, establish either biological relevance or clinical meaningfulness at the individual level.

No symptomatic ARIA-like clinical events were observed in this cohort, including in APOE ε4/ε4 homozygotes. However, routine MRI surveillance was not performed; thus, asymptomatic ARIA-E or ARIA-H cannot be excluded, and clinical observation alone is insufficient to establish the absence of ARIA. This absence of clinically apparent ARIA-like events should, therefore, be regarded as a preliminary, unconfirmed observation rather than evidence of a favorable safety profile relative to anti-amyloid monoclonal antibodies, whose ARIA risk is established through active MRI monitoring in controlled trials that this study did not replicate. Dedicated prospective imaging studies would be required before any comparative safety inference could be drawn.

Limitations: As a preliminary pilot study, limitations include the retrospective single-arm design, small sample size (*n* = 13–16), lack of routine brain MRI for subclinical ARIA detection, and absence of p-tau217 or NfL measurements. The AlzOn Plus assay, while CE-marked under IVDR, has not undergone head-to-head validation against CSF- or PET-based amyloid biomarkers in this cohort, and this study did not establish a longitudinal relationship between this blood biomarker and cerebral amyloid pathology. Blood sampling was performed during routine clinic visits without a fixed time-of-day or fasting protocol; therefore, physiological variation in circulating Aβ oligomer levels related to sampling conditions cannot be excluded. The precise quantitative composition of CNT (specific botanical mass ratios and per-herb dosages) remains proprietary pending intellectual property proceedings. Although the extraction method, dosing frequency, and batch quality-control approach are described in Section 2.2 to support partial reproducibility, full external replication of the exact formulation is not yet possible. The authors intend to disclose complete quantitative details following patent finalization. External cohort comparisons (NACC, ADNI) involve different populations and, in the case of ADNI, a distinct biomarker platform, and, therefore, serve as a directional context only rather than validated quantitative benchmarks. Given the assay’s ~15% within-run coefficient of variation, obtained directly from the manufacturer (Section 2.3), individual-level changes should be interpreted with substantial caution, as differences well beyond the four smallest observed values may fall within analytical variability rather than reflecting a true biological effect. The group-level finding rests primarily on the directional consistency across all 13 patients rather than on the magnitude of any single value (see Discussion). Actual medication adherence was not systematically quantified in this retrospective design. Table 1 reports the prescribed dosing frequency and documented dose modifications, not independently verified adherence. Given the author’s patent and commercial interests in CNT (see Conflicts of Interest), these findings are presented as exploratory and hypothesis-generating rather than confirmatory and should not be interpreted as evidence of therapeutic efficacy.

## 5. Conclusions

In this small, uncontrolled, retrospective pilot cohort, CNT treatment was associated with a mean 15.8% reduction in blood Aβ oligomers at 6 months (Wilcoxon signed-rank test, *p* < 0.001); given the study design, sample size, and assay variability discussed above, this finding should be interpreted as a statistically detectable within-person association rather than confirmed evidence of a clinically meaningful or disease-modifying treatment effect. No clinically apparent ARIA-like events were observed in the four APOE ε4/ε4 carriers, although this observation was not confirmed by MRI. These hypothesis-generating pilot findings provide a rationale for prospective, randomized, controlled trials incorporating neuroimaging and fluid biomarkers within the A/T/N framework.

## Figures and Tables

**Table 1:** Individual prescribed dosing frequency, dose modifications, and concomitant medications (actual adherence was not independently verified).

Patient	Doses/Day	Duration (mo.)	Dose Modification	Concomitant Medication
P1	2 → 1 (after 12 mo)	31	Yes (reduced)	None
P2	3	6	No	Donepezil 5 mg
P3	4 → 3 (after 6 mo)	12	Yes (reduced)	None
P4	3	12	No	None
P5	3	6	No	None
P6	3	6	No	Choline alfoscerate
P7	3	6	No	None
P8	3	6	No	Donepezil 15 mg †
P9	3	12	No	Donepezil 5 mg
P10	3	12	No	Choline alfoscerate
P11	3	12	No	Donepezil 5 mg
P12	3	6	No	Donepezil 5 mg
P13	3	6	No	Donepezil 10 mg
P14	3	12	No	Donepezil 5 mg
P15	3	6	No	None
P16	3	6	No	None

† P8 was excluded from this table’s companion MMSE analysis because hypothyroidism was newly diagnosed (and untreated at the time of blood sampling) at the 6-month assessment, consistent with its exclusion from the primary blood Aβ analysis.

**Table 2:** Patient characteristics and blood Aβ changes.

Patient	Age/Sex	Diagnosis	APOE ε4	Baseline Aβ (ng/mL)	6-Month Aβ (ng/mL)	6-Month % Change	Final Follow-Up (mo.)	Final Aβ (ng/mL)
P1	58/F	Asymptomatic (borderline Aβ)	Not tested	0.86	0.86	0.0%	31	0.56
P2	63/M	EOAD	Not tested	0.72	0.68	−5.6%	6	0.68
P3	53/M	EOAD; APOE ε4/ε4	ε4/ε4	0.96	0.74	−22.9%	12	0.81
P4	73/F	Asymptomatic (borderline Aβ)	Not tested	0.91	0.90	−1.1%	12	0.83
P5	49/F	MCI; APOE ε4/ε4	ε4/ε4	0.96	0.76	−20.8%	6	0.76
P6	57/F	MCI	Not tested	0.78	0.51	−34.6%	6	0.51
P7	76/F	Asymptomatic high-risk	Non-carrier	1.06	0.65	−38.7%	6	0.65
P8 †	73/M	MCI (new-onset hypothyroidism †)	ε4 het.	0.36	0.45	Excl.	6	0.45
P9	59/F	MCI; APOE ε4/ε4	ε4/ε4	0.82	0.63	−23.2%	12	0.68
P10 ‡	63/F	Asymptomatic (ceiling baseline ‡)	Non-carrier	5.00 ‡	1.40	See note ‡	12	0.71
P11	57/F	Asymptomatic high-risk	Non-carrier	1.07	0.85	−20.6%	12	0.64
P12	61/F	MCI	ε4 het.	0.55	0.54	−1.8%	6	0.54
P13	68/F	MCI; APOE ε4/ε4	ε4/ε4	0.44	0.40	−9.1%	6	0.40
P14 ‡‡	84/M	MCI (no pre-treatment baseline ‡‡)	Not tested	—	1.15	N/A	12	0.73
P15	65/F	MCI	Non-carrier	0.73	0.54	−26.0%	6	0.54
P16	71/F	MCI	Non-carrier	0.60	0.59	−1.7%	6	0.59

† P8 excluded: hypothyroidism was newly diagnosed at the 6-month assessment based on incidental laboratory findings; the condition was untreated at the time of blood sampling (no thyroid medication had been prescribed), but the newly identified endocrine disorder itself was considered a potential confounder for interpreting thyroid–amyloid metabolism. ‡ P10: ceiling baseline (5.00 ng/mL = assay maximum), excluded from aggregate statistics. ‡‡ P14: no pre-treatment baseline available. EOAD, early-onset Alzheimer’s disease; MCI, mild cognitive impairment.

**Table 3:** Individual MMSE trajectories in the paired-MMSE subset (*n* = 8).

Patient	Baseline MMSE	6-Month MMSE	Δ	Direction
P2	19	21	+2	Improved
P3	24	22	−2	Worsened
P5	28	26	−2	Worsened
P6	24	28	+4	Improved
P12	21	25	+4	Improved
P13	24	23	−1	Worsened
P14	23 *	25	+2	Improved
P16	26	28	+2	Improved

* P14 baseline MMSE was measured at Chongnoi Korean Medical Clinic (23); a separate score (16) obtained at an outside hospital was not used, to maintain measurement consistency with all other patients. Worsening was defined as any decline in MMSE score from baseline, without a pre-specified minimum threshold.

## Data Availability

The raw data supporting the conclusions of this article will be made available by the authors on request.
